# Detection and Molecular Identification of Phytoplasmas Associated with Potato in Iran

**DOI:** 10.3390/microorganisms14040779

**Published:** 2026-03-30

**Authors:** Ghobad Babaei, Majid Siampour, Assunta Bertaccini

**Affiliations:** 1 Plant Protection Research Department, Chaharmahal and Bakhtiari Agricultural and Natural Resources Research and Education Center, Agricultural Research, Education and Extension Organization (AREEO), Shahrekord 8813657351, Iran; 2 Department of Plant Protection, College of Agriculture, Shahrekord University, Shahrekord 64165478, Iran; 3 *Alma Mater Studiorum*—University of Bologna, Plant Pathology, 40127 Bologna, Italy

**Keywords:** 16SrI-B subgroup, 16SrI-R subgroup, 16SrVI-A subgroup, 16SrXII-A subgroup, potato disease, ‘*Candidatus* Phytoplasma’ species

## Abstract

Potato purple top is a complex phytoplasma disease that poses a serious threat to potato cultivation worldwide. To verify the presence of different phytoplasma strains in potato disease outbreaks in Iran, six major potato-growing provinces in the central and western regions of the country were surveyed, and a total of 270 potato plants, 230 symptomatic and 40 asymptomatic, was sampled. Nested PCR analysis revealed the phytoplasma presence in 45% of symptomatic and 7% of asymptomatic plants. Molecular analysis was performed, analyzing the sequences of the 16S rRNA gene and the ribosomal protein *rp*, *secY*, and *tufB* genes. Four ‘*Candidatus* Phytoplasma’ species were identified in the tested potato samples: ‘*Ca*. P. asteris’ (16SrI-B), ‘*Ca*. P. tritici’ (16SrI-R), ‘*Ca.* P. trifolii’ (16SrVI-A), and ‘*Ca*. P. solani’ (16SrXII-A). The ‘*Ca*. P. solani’ strains were prevalent, occurring in all the surveyed provinces, whereas the ‘*Ca*. P. tritici’ strains were restricted to the Chaharmahal and Bakhtiari province. Phylogenetic and multilocus sequence analyses provided a finer resolution, distinguishing among some of the closely related phytoplasma strains. This study represents the first comprehensive molecular survey of potato-infecting phytoplasmas across a wide geographical region of Iran. The findings will aid studies regarding insect vector(s), pathogen biology, host range and disease management strategies.

## 1. Introduction

Potato (*Solanum tuberosum* L.) represents a critical component of global food security, ranking as the fourth most important crop worldwide [1]. Its global annual production exceeds 383 million tons, with cultivation spread over an estimated 16 million hectares. In Iran, the potato is of considerable agricultural importance, since it is cultivated in more than 80,000 hectares, providing yields around 2.5 million tons in 2023 [2].

Phytoplasmas are cell wall-less bacteria classified under the class *Mollicutes*. These intracellular pathogens inhabit the phloem tissue of plants and are mainly disseminated and transmitted by phloem-feeding insects, including leafhoppers, planthoppers, and psyllids. Phytoplasmas are associated with severe diseases in thousands of plant species, many of which are of substantial economic significance, thereby exerting a profound impact on global agricultural and horticultural production [3]. The symptoms induced in host plants are diverse, including witches’ broom, virescence, phyllody, chlorosis, stunting, and numerous other abnormalities [4]. Phytoplasmas are assigned to the provisional genus ‘*Candidatus* Phytoplasma’ based on the 16S rRNA gene sequence threshold of 98.65%, or alternatively, by genome sequence analysis or analysis of selected housekeeping gene sequences [5]. Sequence and RFLP analysis of the 16S rRNA gene has been also widely used for phytoplasma differentiation. To date, phytoplasmas are distinguished in 16Sr groups based on actual or virtual RFLP analysis of their 16S rRNA with each groupincluding one to numerous subgroups [6].

Multiple phytoplasmas have been associated with diseases in potatoes across the world, and potato purple top (PPT) is a name commonly given to phytoplasma diseases associated with phytoplasma strains belonging to at least eight 16S rRNA groups. These diseases are threatening potato crops in many regions of the world, including North and Central America, Asia, Australia and Europe. A PPT disease associated with the presence of ‘*Ca.* P. solani’ strains is also described as “stolbur” and mainly reported in Europe [7,8,9,10,11,12,13]. Phytoplasma symptoms in potato showing PPT or “stolbur” manifest as purple or yellow leaf discoloration, leafroll, aerial tuber formation, witches’ broom and sprouting in stored tubers, resulting in significant economic losses [9,10]. While some phytoplasma strains within the 16SrI, 16SrVI, and 16SrXII groups exhibit a worldwide distribution, those in16SrXVIII, 16SrV and 16SrIII groups have a restricted geographical area and were mainly, but not only, detected in the American continent and in Asia [10,14,15,16,17,18,19,20,21]. The presence in potato of phytoplasmas belonging to 16SrI (‘*Ca*. P. asteris’), 16SrVI (‘*Ca*. P. trifolii’), and 16SrXII (‘*Ca*. P. solani’) has been also reported in Iran. Preliminary studies suggested that ‘*Ca.* P. solani’ is the phytoplasma prevalent in potato in the country [22,23]. In this study, a multilocus sequence analysis was performed for a detailed characterization of phytoplasmas detected in potato samples collected from a wide geographical area in Iran to verify the presence of genetic diversity. The 16S rRNA, *rp*, *secY*, and *tufB* genes of phytoplasma strains infecting potato in central and western Iran were therefore analyzed.

## 2. Materials and Methods

### 2.1. Sample Collection and DNA Extraction

From summer to autumn during 2022–2024, plant leaves were collected from 270 potato plants (230 symptomatic and 40 asymptomatic), from the main potato-growing regions located in different geographical areas of Iran, including the provinces Isfahan (21 symptomatic and 4 asymptomatic), Chaharmahal and Bakhtiari (120 symptomatic and 16 asymptomatic), Fars (30 symptomatic and 5 asymptomatic), Hamedan (26 symptomatic and 5 asymptomatic), Kurdistan (23 symptomatic and 5 asymptomatic) and Markazi (10 symptomatic and 5 asymptomatic). During these surveys performed in fields of approximately identical surfaces, the samples were collected from eight potato cultivars (Agria, Sante, Raja, Santana, Navita, Burren, Marfona, Diamant). In each province, at least five potato fields were surveyed, and at least five symptomatic plants per field were sampled, except for the Markazi province, where only two symptomatic plants per field were collected. The disease incidence was assessed by calculating the ratio of symptomatic to total plants (asymptomatic and symptomatic) in five randomly selected areas of each field, with each area containing at least 100 plants. Total DNA was extracted from 0.2 g of leaf midribs using a described method [24].

### 2.2. Phytoplasma Detection and Differentiation

The amplification of 16S rRNA gene sequences was conducted using nested PCR with primer pairs P1/P7 [25,26], followed by R16F2n/R16R2 (for detection and RFLP analyses) or R16mF2/R16mR2 (for sequencing) [27]. All PCR amplifications were conducted for 35 cycles in an automated thermal cycler (Auto Q, Quanta Biotech, Surrey, UK). PCR mixtures prepared contained 50 ng of total DNA, 0.4 µM of each primer, and FIREPOL master mix (200 µM of dNTPs, 1.5 mM MgCl_2_) (Solis Biodyne, Tartu, Estonia), in a total volume of 25 µL. The PCR conditions were denaturation at 94 °C for 30 s (4 min in the first cycle), primer annealing at 54 °C for 30 s and primer extension at 72 °C for 75 s (10 min in the last cycle). Each PCR assay included a negative control devoid of DNA template. For nested PCR amplifications, 1 µL of the 1: 20 diluted PCR products from the first amplification was used as a template. PCR products were electrophoresed through a 1.2% agarose gel, stained with ethidium bromide, and visualized under UV light. The R16mF2/R16mR1 amplicons were directly sequenced (using the same primers) in both directions with three replicates.

RFLP analysis was carried out to distinguish the phytoplasma strains detected in the tested potato plants. The amplicons obtained by nested PCR amplification using the primer pairs P1/P7 and R16F2n/R16R2 were digested with restriction enzymes *Alu*I, *Mse*I, *Rsa*I, *Taq*I, *Hpa*II, *Hha*I, *Hae*III, *Hinf*I, *Kpn*I (Fermentas, Thermofisher, Waltham, MA, USA) following the manufacturer’s instructions. The resulting products were then separated using 8% polyacrylamide gel electrophoresis, stained with ethidium bromide 1% solution, and visualized under UV light. Additionally, to assign each phytoplasma to specific 16Sr subgroups, a virtual RFLP analysis of the 16S rRNA gene (trimmed to the R16F2n/R16R2 primers) was conducted using the *i*PhyClassifier online tool (https://plantpathology.ba.ars.usda.gov/cgi-bin/resource/iphyclassifier.cgi (accessed on 20 November 2025)) [28].

### 2.3. Multilocus Gene Sequence Amplification

Nested PCR was employed to amplify specific phytoplasma genomic regions, including the *tufB*, *rp* (*rplV-rpsC*), and *secY* housekeeping gene sequences. A partial *tufB* gene sequence was amplified using the primer pairs Tuf340/Tuf890 followed by Tuf400/Tuf835 [29]. The amplification of the *rp* gene operon was performed using the primer pairs rp (I)FIA/rp(I)RIA [30,31]. Additionally, the primer pair secYF1/SecYR1 [32] was used for amplification of the *secY* gene. All amplifications were performed as described above, with an annealing temperature of 50 °C. The amplicons were sequenced in both directions with the same primers used for the respective amplifications with three replicates. The resulting sequences were assembled using DNAMAN (www.lynnon.com) and compared to the GenBank database using BLASTn version 2.17.0.

### 2.4. Phylogenetic Analysis

The phylogenetic relationship among the phytoplasmas detected in this study and representative phytoplasmas of different 16Sr groups/subgroups for which the sequences of the studied genes were available (Table 1) was examined using the 16S rRNA, *tufB*, *rp*, and *secY* gene sequences. The sequences were aligned using CLUSTALW (https://www.genome.jp/tools-bin/clustalw (accessed on 10 December 2025)), implemented in the MEGA 11 software [33]. Each gene sequence was then used to reconstruct phylogenetic trees using the maximum likelihood method available in MEGA 11. To assess the reliability and support of the branches on each phylogenetic tree, a bootstrap test with 1000 replicates was conducted.

## 3. Results

### 3.1. Symptomatology Study and Phytoplasma Detection

**Figure 1:**
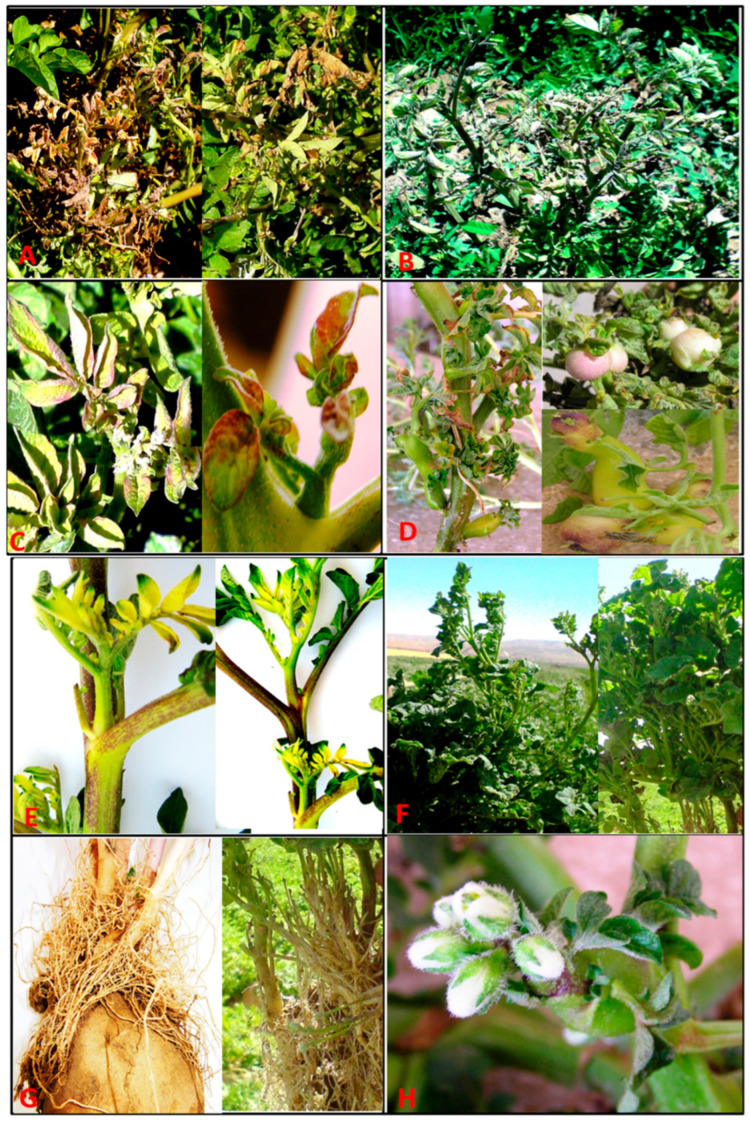
Symptoms observed from different fields across Iran in potato plants infected by different phytoplasma strains. Purple top wilt and necrosis (**A**,**B**), purple leaf and leaf roll (**C**), aerial tuber formation (**D**), leaf yellowing (**E**), proliferation and witches’ broom (**F**), hairy root (**G**) and adventitious flower in a lateral bud (**H**).

A variety of symptoms were observed in potato plants during the field surveys across the surveyed provinces of Iran. These symptoms included purple top, necrosis, dwarfing, aerial tuber formation, hairy root, witches’ broom and proliferation (Table 1, Figure 1).

Notably, purple top was the prevalent symptom, occurring in more than 50% of the symptomatic plants either alone or in combination with other symptoms. The incidence of symptomatic potato plants in the surveyed fields was estimated as ranging from 2% to 40%. The presence of phytoplasmas in the potato plant samples was evaluated by nested PCR using primers P1/P7 followed by R16F2n/R2. Of the symptomatic potato plants tested, 103 (45%) were positive for phytoplasma presence. Nested PCR analysis also revealed a phytoplasma presence in 7% of the asymptomatic plants. 

### 3.2. Phytoplasma Identification

**Figure 2:**
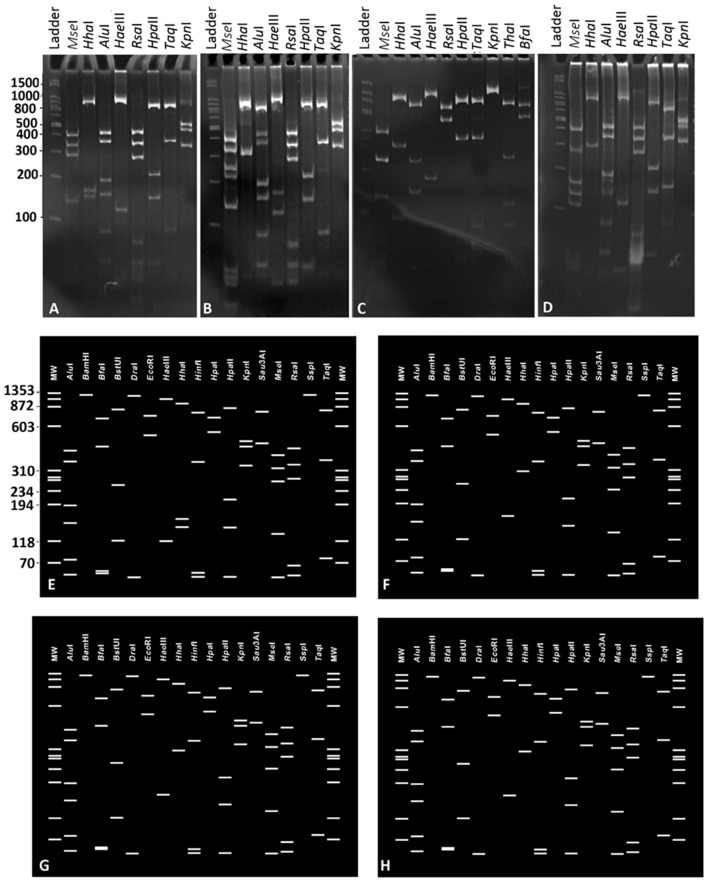
Actual (**A**–**D**) and virtual (**E**–**H**) RFLP patterns of R16F2n/R2 amplicons and sequences, respectively, from four phytoplasma strains detected in diseased potato plants in Iran. The phytoplasma strains are PPT1 (**A**,**E**), PPT2 (**B**,**F**), PPT3 (**C**,**G**) and PPT4 (**D**,**H**).

The 1250 bp R16F2n/R16R2 amplicons were analyzed by RFLP to differentiate the phytoplasmas and select representative strains for sequencing. In total, 16S rDNA RFLP patterns from 60 of the 103 phytoplasma-positive samples were compared, with 5–20 samples randomly analyzed from each province. The number of selected samples was based on the frequency of phytoplasma positive detections in each province, also ensuring the inclusion of plants exhibiting all the varieties of symptoms collected. The RFLP analysis identified four distinct patterns among the analyzed phytoplasmas (Figure 2). A total of 15 strains representing each distinct RFLP pattern representative from each province was selected for 16S rRNA gene sequencing. A 1.25 kbp DNA fragment of the 16S rRNA gene was obtained from all these samples after sequencing using nested PCR with primers P1/P7, followed by R16mF2/R16mR2.

The analysis of the 16S rRNA gene sequences of these 15 phytoplasmas distinguished four unique sequences corresponding to the results obtained from the RFLP analyses. These sequences were deposited in GenBank under the accession numbers PX953017-PX953020. The 16S rRNA gene sequences of these four strains (PPT1-PPT4) were compared with the reference strains of ‘*Ca*. Phytoplasma’ species [5]. The strain PPT1 showed 99.92% (1 GAP and 1 SNP) identity to ‘*Ca*. P. asteris’, 16SrI-B. The strain PPT2 had 99.92% (1 SNP) identity to ‘*Ca*. P. tritici’ (16SrI-C/R). The strain PPT3 had 99.68% (4 SNPs) identity to ‘*Ca*. P. trifolii’ (16SrVI-A), and the strain PPT4 showed 99.68% (4 SNPs) identity to ‘*Ca.* P. solani’ (16SrXII-A) (Table 2).

The virtual RFLP patterns obtained for each strain were consistent with the corresponding actual RFLP profiles (Figure 2); in particular, strains PPT1 and PPT2 were assigned to the subgroups 16SrI-B and 16SrI-R (reclassified as 16SrI-C rrnA), respectively, each with a 100% similarity coefficient. Likewise, strains PPT3 and PPT4 were assigned to the subgroups 16SrVI-A and 16SrXII-A, respectively, with a 100% similarity coefficient.

### 3.3. Phytoplasma Prevalence

Among the collected samples, the highest percentage of phytoplasma detection was recorded in the provinces of Isfahan, Fars, Markazi, Hamedan, Chaharmahal and Bakhtiari, and Kurdistan respectively (Table 1). It is noteworthy that the variation in sample sizes among provinces may have influenced the infection rates calculated for each region. Furthermore, RFLP analyses indicated that phytoplasmas belonging to the subgroups 16SrXII-A, 16SrI-B, 16SrVI-A, and 16SrI-C/R accounted for approximately 64%, 24%, 10%, and 2% of the infected plants, respectively (Table 1). Phytoplasmas of subgroup 16SrXII-A were the most common and were detected in all the six provinces surveyed. In contrast, strains of the 16SrI-C/R subgroup were found only in a few potato samples from Chaharmahal and Bakhtiari province. Although the plants tested showed a variety of symptoms, no clear association was observed between symptom types and ‘*Ca*. Phytoplasma’ strains (ribosomal subgroups).

### 3.4. Phylogenetic Analysis of 16S rRNA Gene

**Figure 3:**
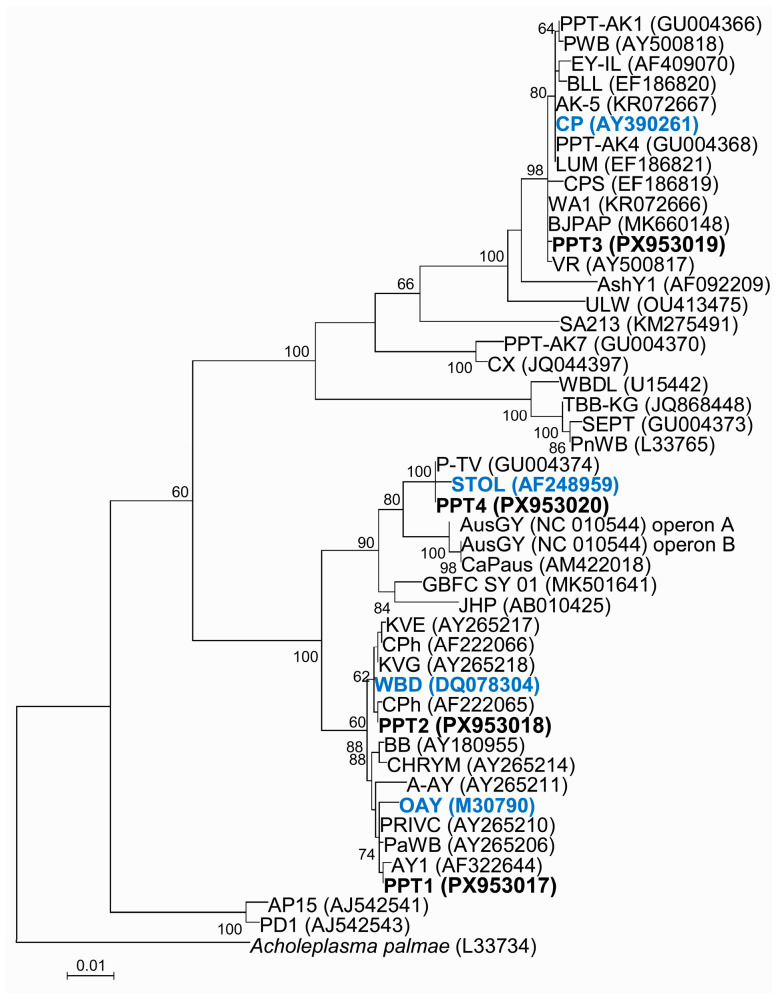
Phylogenetic relationships among Iranian potato phytoplasma strains (PPT1-PPT4) and phytoplasmas belonging to different 16Sr groups/subgroups based on analysis of the 16S rRNA gene sequence using the maximum likelihood method in MEGA 11. The robustness of the tree topology was assessed through a bootstrap test with 1000 replicates, and bootstrap support values of >60 (expressed as percentages) are indicated adjacent to branches. The 16S rRNA gene sequence of *Acholeplasma palmae* was used as an outgroup to root the tree. Scale bar represents 0.01 nucleotide substitutions per site. Table 1 reports the details of the phytoplasma strains. In bold are phytoplasmas from potato detected in this work, in blue bold are the reference ‘*Ca.* Phytoplasma’ strains: OAY = ‘*Ca*. P. asteris’; WBD = ‘*Ca*. P. tritici’; STOL = ‘*Ca*. P. solani’ and CP = ‘*Ca*. P. trifolii’.

A maximum likelihood phylogenetic tree was constructed using the 16S rRNA gene sequences from this study, along with more than 40 phytoplasma strains from different 16Sr groups (Figure 3, Table A1). The analysis revealed that strains PPT1 and PPT2 clustered with strains of the subgroups 16SrI-B and 16SrI-C/R, respectively. The phylogenetic tree indicated that the strain PPT1 had a closer relationship with OAY, reference strain of ‘*Ca*. P. asteris’, while PPT2 was more closely related to WBD, reference strain of ‘*Ca*. P. tritici’. Furthermore, strains PPT3 and PPT4 clustered with strains in subgroups 16SrVI-A and 16SrXII-A, respectively. Thus, the strain PPT3 was found to be closely related to CP, reference strain of ‘*Ca*. P. trifolii’, whereas PPT4 showed a close phylogenetic relationship to STOL, reference strain of ‘*Ca*. P. solani’. Overall, these results confirm the phytoplasma classification based on 16S rRNA gene sequence identity percentages.

### 3.5. Multilocus Sequence Analyses

DNA fragments of approximately 1250 bp, 1200 bp, and 400 bp, corresponding respectively to the gene sequences *secY*, *rp* (*rplV rpsC*), and *tufB*, were amplified using nested PCR. These fragments were obtained from phytoplasma strains PPT1 (16SrI-B), PPT2 (16SrI-C/R), PPT3 (16SrVI-A) and PPT4 (16SrXII-A). The nucleotide sequences of each strain were deposited in GenBank under accession numbers for *rp* (*rplVrpsC*): PX981416-PX981419, *secY*: PX964288-PX964291, and *tufB*: PX964292-PX964295 (Table A2). Analysis of pairwise sequence identities among these strains revealed that strains PPT1 and PPT2 exhibited the highest identity: 98.04% for rp, 97.57% for *tufB*, and 95.45% for *secY*. In contrast, the lowest sequence identity was observed between strains PPT3 and PPT4, which showed identities of 66% for *rp*, 76.5% for *tufB*, and 74% for *secY*. BLASTn analysis of the gene sequences from the four phytoplasma strains (PPT1, PPT2, PPT3, and PPT4) indicated that the majority of each gene sequence had more than 99% identity with sequences from several phytoplasma related strains available in GenBank. Specifically, strains PPT1 and PPT2 show a higher identity to ‘*Ca*. P. asteris’ and ‘*Ca*. P. tritici’ strains, respectively. Strains PPT3 and PPT4 show a higher identity to ‘*Ca*. P. trifolii’ and ‘*Ca*. P. solani’ strains, with low values of 97.51% and 95.86% to the reference strains only for the *rplV-rpsC* gene (Table 2). In this regard, BLASTn revealed that PPT1 had sequence identities of 99.5–100%, 99.2–100%, and 100% for the *rp*, *secY*, and *tufB* genes respectively, when compared to ‘*Ca*. P. asteris’ (16SrI-B) strains from different countries. PPT2 exhibited the sequence identities of 96–100%, 99.6–100% and 100% for the *rp*, *secY*, and *tufB* genes respectively, when compared to strains of ‘*Ca*. P. tritici’ from different countries. Regarding PPT3, the *rp*, *secY*, and *tufB* gene sequences showed sequence identities of 97.4–99.6%, 95.2–99.2% and 97.7–100%, respectively, to ‘*Ca.* P. trifolii’. Similarly, the *rp*, *secY*, and *tufB* gene sequences of the strain PPT4 showed, respectively, 99.4–100%, 99.2–100% and 99.7–100% identity to several strains of ‘*Ca*. P. solani’ (16SrXII-A) from various countries. The sequence identity analysis revealed that the *secY* gene exhibited the highest resolving power in distinguishing phytoplasma strains within the ‘*Ca*. P. trifolii’ strains (16SrVI group). The PPT3 strain exhibited a *secY* sequence identity of 99.2% with the VR strain and 95% identity with strain LUM, both belonging to the 16SrVI group.

**Figure 4:**
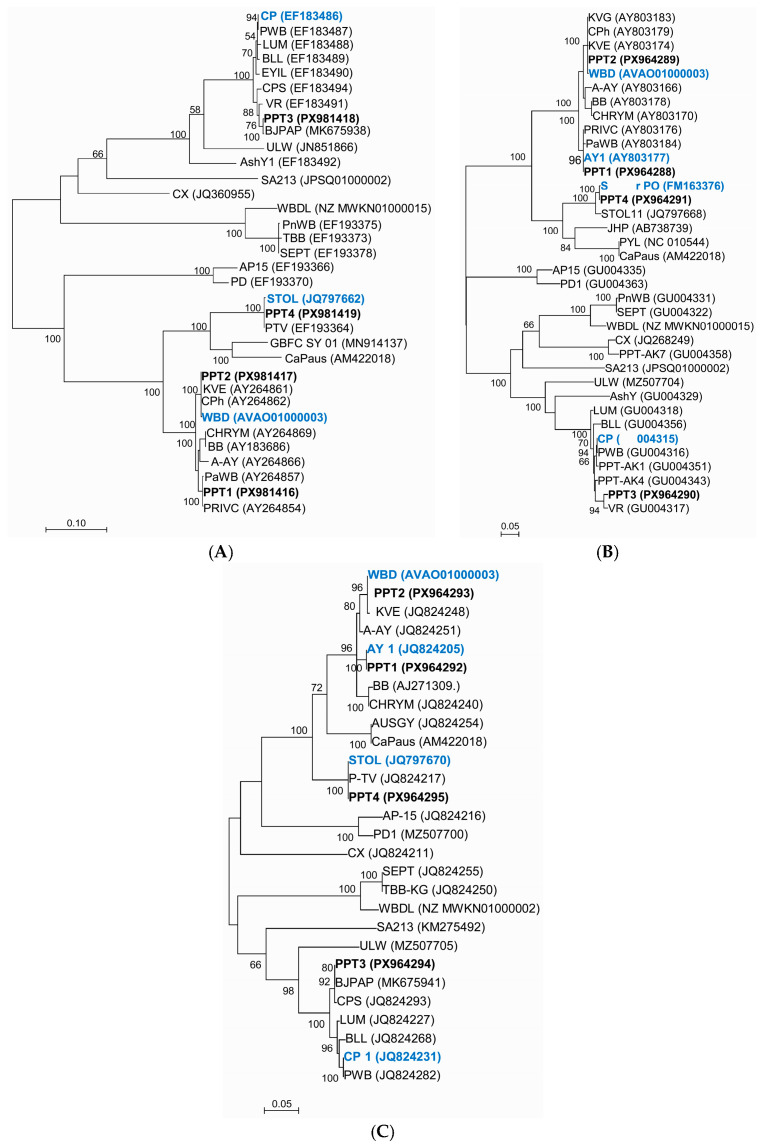
Phylogenetic relationships among the Iranian potato phytoplasma strains (PPT1-PPT4) and strains of phytoplasmas belonging to different 16Sr groups/subgroups based on analysis of the *rp* (*rplV-rpsC*) (**A**), *secY* (**B**) and *tufB* (**C**) gene sequences using the maximum likelihood method in MEGA 11. The robustness of the tree topology was assessed through a bootstrap test with 1000 replicates, and bootstrap support values of >60 (expressed as percentages) are indicated adjacent to branches. Scale bar represents 0.10 or 0.05 nucleotide substitutions per site. Table 1 reports the details of these phytoplasma strains. In bold are marked the phytoplasmas from potato detected in this work, in blue bold are the reference ‘*Ca.* Phytoplasma’ strains.

Phylogenetic trees were reconstructed using the *rp*, *secY*, and *tufB* gene sequences to verify the phylogenetic relationships among the potato phytoplasma strains of this study and other selected phytoplasma strains (Figure 4). The phylogenetic position of the Iranian phytoplasmas was similar across all reconstructed trees. In each of the three phylogenetic trees, PPT1, PPT2, PPT3, and PPT4 clustered with their respective ‘*Ca*. Phytoplasma’ reference strains. The sequence variability of these housekeeping genes is higher than that of the 16S rRNA gene; then, the phylogenetic trees generated using these genes confirmed, with high resolution power, their molecular identification.

## 4. Discussion

Diverse phytoplasmas from multiple 16Sr groups/subgroups have been reported, which poses a threat to potato production in several areas of the world [7,8,9,10,11,12,13,14,15,16,17,18,19,20,21,34,35,36,37,38,39]. This study provides a detailed analysis of the prevalence and genetic variability of phytoplasmas in potato plants across central and western Iranian areas. Four distinct ‘*Ca*. Phytoplasma’ were detected, and this is the first report of ‘*Ca*. P. tritici’ in potato in Iran and of ‘*Ca*. P. trifolii’ in this area of Iran. In Iran, phytoplasmas of 16SrI, 16SrVI, and 16SrXII groups were already reported in potatoes showing mainly purple top symptoms [22,23]. Moreover, the analysis of 16S rRNA gene sequences of potato phytoplasmas from Iran deposited since 2011 in the GenBank database assigned theose strains to the ‘*Ca*. P. asteris’ subgroup 16SrI-B (accession number FJ427297), to the ‘*Ca*. P. solani’, subgroup 16SrXII-A (accession number EU661607), and to the ‘*Ca*. P. trifolii’ subgroup 16SrVI-D (accession number FJ427295), further corroborating the present findings and also indicating persistence of the same phytoplasma strains over time. Moreover, some ‘*Ca*. P. trifolii’ strains showing 99.12% and 99.44% identity to the reference strain (GenBank accession numbers FJ427296 and EU649681) could not be enclosed within any of the reported subgroups, suggesting that they may represent slowly different phytoplasma lineages. However, it must be outlined that, since these bacteria are still difficult to culture [40], in case of the presence of mixed infection or of populations, it cannot be ruled out that specific gene fragments could be amplified from different genotypes producing the heterogeneity observed in some of the sequences.

Although many symptomatic potato plants were infected with phytoplasmas, 55% of the samples tested were negative. This discrepancy may reflect uneven pathogen distribution and the fact that PCR-based detection of microorganisms is not entirely reliable, primers may fail to bind properly due to sequence variations and false positives from other microbes may also occur [41]. The influence of environmental factors or agricultural practices such as the use of molecules that influence the plant hormone balance in symptom induction could also have a role in finding negative results from symptomatic potato samples [42]. A small proportion of asymptomatic plants (7%) was also phytoplasma-positive, likely due to the presence of an incubation period before the development of symptoms. Similarly, Santos-Cervantes et al. [10] reported that 15.1% of asymptomatic tested potato samples from Mexico were positive for phytoplasmas, Moreover, the presence of ‘*Ca*. P. tritici’, ‘*Ca*. P. solani’, ‘*Ca*. P. australasiaticum’ and ‘*Ca*. P. mali’ was similarly detected in asymptomatic shoots from seed potatoes grown under a greenhouse in Italy [34].

The specific contribution of non-phytoplasma factors to potato disease development remains therefore to be further elucidated. However, surveys performed in Iran revealed that weeds and crops surrounding potato fields were phytoplasma-infected. ‘*Ca*. P. solani’ (16SrXII-A) and both ‘*Ca*. P. solani’ and ‘*Ca*. P. trifolii’ were detected in a number of plant species [43,44,45,46,47,48]. An unidentified weed was also infected with 16SrI-R phytoplasmas. These findings suggest that a wide range of plant species may contribute to the spread of phytoplasmas in potato fields in Iran.

The ‘*Ca*. Phytoplasma’ strains identified in this study, as belonging to subgroups 16SrXII-A, 16SrI-B, 16SrVI-A, and 16SrI-C/R, are reported as having wide host range and worldwide distribution. Aster yellows phytoplasmas assigned to 16SrI-B, 16SrI-R (formerly known as 16SrI-C), and 16SrI-A are associated with diseases in more than 80 plant species worldwide and are transmitted by about 30 insect vector species [30]. Similarly, phytoplasmas of subgroups 16SrXII-A (‘*Ca*. P. solani’) and 16SrVI-A (‘*Ca*. P. trifolii’) were reported from different countries around the world, infecting a wide variety of plant host species [43,44].

The high prevalence of ‘*Ca*. P. solani’ and ‘*Ca*. P. asteris’ strains highlights their adaptability to potato-growing conditions in Iran [45,46]. This may be due to their broad host range or the presence of diverse and effective insect vectors transmitting these phytoplasma strains in agreement with the worldwide spread of these phytoplasma strains. Lee et al. [30] reported that phytoplasmas of 16SrI-B (‘*Ca*. P. asteris’) constitute the largest and most diverse group within the aster yellows phytoplasmas. According to the findings of this study, the ‘*Ca*. P. solani’ strains were prevalent in all the surveyed provinces, consistently with previous findings indicating a higher incidence of these phytoplasmas in potato plants compared to ‘*Ca*. P. asteris’ and ‘*Ca*. P. trifolii’ in Iran [16].

These phytoplasmas have been detected in Iran for many years, associated with diseases in various plant host species [47]. Globally, they have been associated with a wide range of diseases, transmitted by multiple insect vectors, and were reported in wide geographic areas, showing a strong capacity of adaptation to a diverse array of plants and insect hosts [9,12,43]. Previous studies indicated that strains of ‘*Ca.* P. trifolii’ are transmitted by the leafhopper *Circulifer tenellus* in Iran [48]. Due to its polyphagous behavior and wide dispersal, this insect vector may also play a role in the transmission of the strains infecting potatoes. In contrast, while ‘*Ca*. P. tritici’ was reported in Iran, particularly in Chaharmahal and Bakhtiari province [49,50], only a few potato plants in this province were found to be infected with this phytoplasma. This finding suggests that the transmission of this phytoplasma to potato plants may have resulted from sporadic transmission by still unknown insects. Further investigation is needed to identify insect vectors and reservoir host plants, which will be helpful in better understanding the epidemiology of phytoplasma diseases in the potato-growing areas of Iran.

Multilocus gene sequence analyses on *rp*, *tufB* and *secY* genes may provide a higher resolution power than 16S rRNA to differentiate closely related phytoplasmas [31,51]. Through this approach, it was found that the 16SrVI-A phytoplasmas in this study are closer to the BJPAP or VR strains than to the BLL or CP strains (all belonging to 16SrVI-A). According to *secY* RFLP-based analyses, Lee et al. [30] differentiated 10 strains of the 16SrVI-A phytoplasmas into seven genotypes, highlighting the presence of genetic diversity of the phytoplasmas in this subgroup. The PPT-3 strain, characterized in this study as belonging to the 16SrVI-A subgroup, exhibited a higher (though still distinguishable) *secY* gene sequence identity to the VR strain genotype, corresponding to the vinca virescence phytoplasma from California, USA (GenBank accession number GU004317). Among the genes analyzed, *secY* demonstrated the greatest diversity and resolution power for differentiating closely related strains, in agreement with previous reports [30]. Sequencing less conserved genes such as *secY* and *rp* from additional phytoplasma strains could provide more comprehensive insights into the population diversity of potato phytoplasmas in Iran.

These findings show that phytoplasma-infected potato plants display various symptoms, even when the same cultivars grown in a single field are infected by molecularly indistinguishable phytoplasma strains. For example, in a field in the Caharmahal and Bakhtiari province, plants of the cultivar Agria infected with ‘*Ca*. P. asteris’, 16SrI-B, displayed proliferation symptoms without purple top, whereas others of the same cultivar in the same field developed purple top symptoms without proliferation. Similar observations were reported in studies by Wei et al. [52], where two variants of 16SrVI phytoplasmas were associated with distinct symptoms in tomato: big bud and witches’ broom. Using the SNP analysis of the 16S rRNA gene, the two closely related phytoplasma strains were distinguished. However, in the present study, the phytoplasma strains of the 16SrI-B group associated with the two different symptoms could not be distinguished through SNP analysis of the 16S rRNA gene or of the other housekeeping gene sequences. Therefore, based on the sequence comparison of the studied gene fragments, no firm conclusions could be drawn about differences in virulence since genotypes with different pathogenicity are indistinguishable with the currently used methods. Sequence analysis of pathogenicity-related genes, including specific effector proteins, could be helpful to distinguish closely related phytoplasma strains from potatoes.

## 5. Conclusions

The findings of this study indicate that diverse ‘*Ca*. Phytoplasma’ strains are present in potato in Iran. Verifying the presence of genetic variability in phytoplasma strains from wider areas and identifying their insect vectors will be helpful for a more comprehensive understanding of the potato-associated phytoplasma diseases’ epidemiology in Iran. This study allowed the detection of phytoplasmas in potato plants showing purple top, aerial tuber formation and yellowing symptoms. Four ‘*Ca*. Phytoplasma’ strains from potato plants were identified based on 16S ribosomal sequences confirmed by multigene sequence analysis and differentiated by RFLP analyses in the subgroups 16SrI-B, 16SrI-C/R, 16SrVI-A and 16SrXII-A.

## Figures and Tables

**Table 1:** Symptom characteristics, phytoplasma detection and identification in different potato cultivars from six provinces in Iran.

Province	Main Symptoms	Positive/Total Tested (%)	Phytoplasma Subgroup (Number)	Potato Cultivars
Chaharmahal and Bakhtiari	Little leaf, proliferation, aerial tuber, purple top, yellowing, big bud, phyllody, dwarfing, hairy root	38/136 (27.9)	16SrI-B (11), 16SrI-R (2), 16SrXII-A (20), 16SrVI-A (5)	Agria, Sante, Raja, Santana, Navita, Burren, Marfona, Diamant
Fars	Wilting, root rot, aerial tuber, purple top, yellowing	16/35 (45.7)	16SrI-B (5), 16SrXII-A (11)	Agria, Burren, Marfona
Hamedan	Little leaf, aerial tuber, purple top, yellowing	12/31 (38.7)	16SrI-B (3), 16SrXII-A (9)	Agria, Sante, Burren, Santana, Marfona
Isfahan	Proliferation, aerial tuber, purple top, yellowing, dwarfing	16/25 (64.0)	16SrI-B (4), 16SrXII-A (9), 16SrVI-A (3)	Agria, Burren, Marfona, Diamant
Kurdistan	Little leaf, proliferation, aerial tuber, purple top	9/28 (32.1)	16SrXII-A (7), 16SrVI-A (2)	Agria, Marfona
Markazi	Aerial tuber, purple top, yellowing, dwarfing	12/15 (80)	16SrI-B (2), 16SrXII-A (10)	Agria, Sante, Burren

**Table 2:** Percentage of identity of the studied genes of the potato phytoplasma strains to the respective reference strain sequences from Bertaccini et al. [5].

Phytoplasma Strain/Reference Strain (GenBank Accession Number of 16S rRNA Gene)	16S rRNA Gene	*rpl-rps3* Gene	*secY* Gene	*tufB* Gene
PPT-1/‘*Ca*. P. asteris’ (PX953017/AP006628)	99.92%	99.48%	99.36%	100%
PPT-2/‘*Ca*. P. tritici’ (PX953018/DQ078304)	99.92%	100%	99.84%	100%
PPT-3/‘*Ca*. P. trifolii’(PX953019/AY390261)	99.68%	97.51%	95.86%	100%
PPt-4/‘*Ca*. P. solani’ (PX953020/AF248959)	99.68%	100%	99.59%	100%

## Data Availability

The data presented in this study are openly available in GenBank NCBI at https://www.ncbi.nlm.nih.gov/genbank/, reference number PX 953017, PX953019, PX953019, PX953020; PX981416, PPX981417,X981418, PX981419; PX964288, PX964289, PX964290, PX964291; PX964292, PX964293, PX964294, PX964295.

## Abbreviations

The following abbreviations are used in this manuscript:

‘*Ca*. P. asteris’	‘*Candidatus* Phytoplasma asteris’
‘*Ca*. P. solani’	‘*Candidatus* Phytoplasma solani’
‘*Ca*. P. trifolii’	‘*Candidatus* Phytoplasma trifolii’
‘*Ca*. P. tritici’	‘*Candidatus* Phytoplasma tritici’
‘*Ca*. Phytoplasma’	‘*Candidatus* Phytoplasma’
BJPAP	*Brassica juncea* phyllody
BLL	Brinjal little leaf
CP	Clover proliferation
DNA	Deoxyribonucleic acid
PCR	Polymerase chain reaction
PPT	Potato purple top
RFLP	Restriction fragment length polymorphism
rRNA gene	Ribosomal ribonucleic acid
SNP	Single-nucleotide polymorphism
USA	United States of America
UV	Ultraviolet
VR	Vinca virescence

## Appendix A

**Table A1:** Phytoplasma strains, associated disease, country of origin, subgroup affiliation and accession numbers of 16S rRNA, *rp*, *tufB* and *secY* genes.

				GenBank Accession Numbers			
Strain	Associated Disease	Country	16Sr Subgroup	16S rRNA	*rp*	*TufB*	*secY*
BB	Tomato big bud	USA	16SrI-A	AY180955	AY183686	AJ271309	AY803178
CHRYM	*Chrysanthemum* yellows	Germany	16SrI-A	AY265214	AY264869	JQ824240	AY803170
OAY	*Oenothera* phytoplasma	USA	16SrI-B	M30790			
PRIVC	Primrose virescence	Germany	16SrI-B	AY265210	AY264854		AY803176
AY (AY-1)	Maryland aster yellows	USA	16SrI-B	AF322644		JQ824205	AY803177
KVE	Clover phyllody	France	16SrI-C	AY265217	AY264861	JQ824248	AY803174
KVG	Clover phyllody	Germany	16SrI-C	AY265218			AY803183
PaWB	Paulownia witches’ broom	Taiwan	16SrI-D	AY265206	AY264857		AY803184
A-AY	Aster yellows apricot	Spain	16SrI-F	AY265211	AY264866	JQ824251	AY803166
CPh	Clover phyllody	Canada	16SrI-R	AF222065	AY264862		AY803179
WBD	Wheat blue dwarf	China	16SrI-R	DQ078304	AVAO01000003	AVAO01000003	AVAO01000003
PnWB	Peanut witches’ broom	Taiwan	16SrII-A	L33765	Ef193375		GU004331
SEPT	Sesame phyllody	Thailand	16SrII-A	GU004373	EF193378	JQ824255	GU004322
WBDL	Witches’ broom of lime	Oman	16SrII-B	U15442	NZ_MWKN01000015	NZ_MWKN01000002	NZ_MWKN01000015
TBB	Tomato big bud	Australia	16SrII-D	JQ868448	EF193373	JQ824250	
CX	Peach X disease	Canada, USA	16SrIII-A	JQ044397	JQ360955	JQ824211	JQ268249
AKpot7	Potato purple top	USA	16SrIII-F	GU004370			GU004358
ULW	Elm yellows	France	16SrV-A	OU413475	JN851866	MZ507705	MZ507704
LUM	Lucerne virescence	France	16SrVI	EF186821	EF183488	JQ824227	GU004318
CP/CP-1	Clover phyllody	Canada	16SrVI-A	AY390261		JQ824231	GU004315
WA1	Columbia Basin potato purple top	USA	16SrVI-A	KR072666			
AK-5	Alaska potato witches’ broom	USA	16SrVI-A	KR072667			
PPTAK1	Potato purple top	USA	16SrVI-A	GU004366			GU004351
PWB	Potato witches’ broom	Canada	16SrVI-A	AY500818	EF183487	JQ824282	GU004316
PPT-AK4	Potato purple top	USA	16SrVI-A	GU004368			GU004343
VR	Vinca virescence	USA	16SrVI-A	AY500817	EF183491		GU004317
BJPAP	Brassica juncea phyllody	China	16SrVI-A	MK660148	MK675938	MK675941	
EYIL	Illinois elm yellows	USA	16SrVI-C	AF409070	EF183490		
CPS	*Catharanthus* phyllody	Sudan	16SrVI-C	EF186819	EF183494	JQ824293	
BLL	Brinjal little leaf	India	16SrVI-D	EF186820	EF183489	JQ824268	GU004356
AshY1	Ash yellows	USA	16SrVII-A	AF092209	EF183492		GU004329
SA213	Almond witches’ broom	Lebanon	16SrIX-B	KM275491	JPSQ01000002	KM275492	JPSQ01000002
AP15	Apple proliferation	Italy	16SrX-A	AJ542541	EF193366	JQ824216	GU004335
PD1	Pear decline	Italy	16SrX-C	AJ542543	EF193370	MZ507700	GU004363
STOL	“Stolbur”	Serbia	16SrXII-A	AF248959	JQ797662	JQ797670	JQ797668
Stolbur PO	Vinca “stolbur”	France	16SrXII-A				FM163376
PTV	Tomato “stolbur”	Italy	16SrXII-A	GU004374	EF193364	JQ824217	
PYL	*Phormium* yellow leaf	New Zealand	16SrXII-B	U43570			NC_010544
AUSGY	Australian grapevine yellows	Australia	16SrXII-B	NC_010544		JQ824254	
CaPaus		New Zealand	16SrXII-B	AM422018	AM422018	AM422018	AM422018
JHP	*Hydrangea* phyllody	Japan	16SrXII-D	AB010425			AB738739
GBFC_SY_01	Strawberry yellows	Lithuania	16SrXII-E	MK501641	MN914137		

## Appendix B

**Table A2:** Sequences of phytoplasma strains *rp*, *tufB* and *secY* genes.

Phytoplasma Strain	s*ecY* Gene	*rp* Gene	*tufB* Gene
PPT1	AAATTAGTTTTAGGCAATCGTAAATTAATGTTTCAAATCTTTTTTACCTTATTTATTATTTCTATTGTTTGTTTAGGAACTTCTTGGCCTCTTCCTTTTATTAATACTAAATCCCTTATTTGTCCAAACTTTTTGGGGTTTTTTCCATAAATGCTGGTACTCTTTTTGGATTGGGAATCACTCCTTACATCACTGCTTCCATTGTAGTGCAATTTTTGCAAAAACTTCTTCCTATTTGTCGCGAATGGAAAGACCAAGGACAAATGGGCAAACGCAAACTTAATCTTTTAACACGTAGTCTTGCCTTATTGTTTGCTTTTGGGCAATCTTTTGCTTTTTTGAACAGTTATTCAAAACTTTTTGTCACATCAATAAGTACAAGCCAACTGTTTTTGTTAGCTTTAATTGCTACTGCAGGAGTTGCTATTTTAATTTGGTTTGCTGACCTTATCAATTCCAAAGGTATTGGAAACGGGACTTCTATTTTAATTGTTGTTTCGATGAGCCACAGCCTAATTAATCTATTTGTAAATCTGAACGAATCATATTTATCTCAAAACAATTTTTTAACTTTGAAAACTTTTAATTTTGCATGTATTGTTCTTTTACTTCTCTTATTTTTAATTTTTACTGTAGTTGTGCAAATAACATCTTTAAAAATACCTATCAATTATGCGCGCAATCAAGTGCAAGGGAAAAGCTACATTCCATTAAAAATTAATAGTGCAGGAGTTATGCCAGTTATTTTGGCATCTGCTTTATTGCAACCTTTCCAGATGTTAGCAGGAGTTATTGGGAATACAAAATTTACAGAATTAGTAGATTTTTTTGCCAAAACTAACTTTCCTGGAAACCAAATTAACTTTTTTGCCATAGGCTTTTTAGTCTTGTTAGTAATTGTTTTTTCTTTCTTTTCTGCTTTTATGAATGTCAATCCTGAAGATATTTCAGAACATTTATCCAAACAAGATGCCTATATTGCAGGTTTAAGACCAGGTGAACAAACTACTCGTTATTTAGCTAATACCTTATTTAAAATCACTGTTTTAGGAACTGTTTTTATTGCTGCTCTTGTTGTAACACCTATTCTTATGGAACATTTTTTAGGTTTGAAAGATATGAAATTAGGAGGAACCAGTTTGCTTATTATTGTTAGTGTAGCCCTTGAAACTATCCAACGCATCAAAGCTACTGCCAACAAAAAAGAATATCAAAAATTATTTTAATTAAGCAAACAAGACAATATGATACTAAT	CTTACCGCGGACACAACAAAAAAGACAAAAAAATTCAAAAAAAATAAAATAATGGGAAGGAATAACTATGGAAACCAAAAACGCCAAAGCGATTGCTAGAAAAGTTTCAATCGCCCCTCGAAAAGCACGTTTAGTTGTTGATTTAATTCGAGGAAAAAATATTGCACAAGCTCAAGCCATTTTAACTTTTACCCCTAAAGTAGCTGCTCCCGTTATTTTAAAACTTTTAAACAGTGCTGTTTCCAATGCTGTTAATAATTTAAAATTAAACCGCGAACAACTTTATGTTAAAGAAGTTTTTGTCAACGAAGGTTTGCGTTTAAAACGTATGTTTCCAAGAGCTAAAGGTTCTGGTGATATGATTAAAAAAAGAACCAGCCACATTACTTTAGTAATAACTTCTAGCACAAACTTGCAAACATCAAAGGAGGAAGAACAAAGTGGGTCAAAAAACTAATCCTAACGGCTTAAGATTAGGCATTATTAGAACTTGGGAATCTCAATGGTGTGTTAATGATAAAGAAATTCCTAATTTAATTAAAGAAGATTTTTTAATTCGTAAACTAATCAATAATTTTACTAAAAAAAGTGCTATCAGTCAAATTGACATTGAACGCCTAAAAGAAAAAAATAAAAACCGTATCACTATTTCTGTCCACACCGCTAAACCAGGCGTTATTATTGGAAAAGATGGCGATACACGCAACAAATTAGTTGCCAAACTCAAAGAACTTACCCAAAAAGACGTTAATCTTAACGTGTTAGAAGTTAAAAACTCTGATAAAATCGCTTTATTAATTGCTCAAAATATGGCTGAACAACTAGAAAATCGTATGTTTTTCCGCCGTGTTCAAAAAATGGCAATCCAAAAAGCCCTAAAAGCTGGTGCCAAAGGAGTAAAAACTTTAATTTCTGGTCGTTTGGGTGGTGCTGAAATAGCTCGTAGCGAAGGACATGCCGAAGGCAGAGTTCCTCTACACACTCTAAGAGCAGACATCGATTACGCTGCTGTTGAAGCTCACACTACTTATGGAGTTTTAGGAATTAAAGTATGGATTTTCCACGGTGAAGTTTTACCAGGACAAACCATTCTAGACACTAGAAAACCGTTTGCTTCCCAATCTTCTAACACTCCTAACAGACGCCCTCGCAAT	TGCTCACGTTGACTGTCCTGGACACGCCGATTATGTTAAAAACATGATTACTGGTGCTGCTCAAATGGATGCTGCTATTTTAGTTGTTTCTGGTGCAGATAGTGTTATGCCTCAAACTAGAGAACACATTTTATTAGCGCGCCAAGTTGGTGTTCCAAAAATTGTTGTTTTCTTAAATAAATGTGATCTTTCCCCAGACGAACAAATTTTAGAATTAGTAGAAATGGAAGTTCGTGAATTATTATCTCAATACGATTTCCCAGGAGACGACATTCCTGTTATTAGAGGATCTGCATTAAAAGCTTTAGAAGGTGACGCTCATTACGTTGCTCAAGTAAACGAATTAATCGAAACTCTAGACACCTACATTGAAGACCCGGTGCGCGAAGTTGACAAACCTTTCTTAATGCCT
PPT2	AAATTAGTTTTAGGCAATCGTAAATTAATGTTTCAAATATTTTTTACCTTATTTATTATTTCTATTGTTTGTTTAGGAACTTCTTGGCCTCTTCCTTTTATTAATTCTAAATCTCTTGATTTGTCAAAACTTTTTGGGGTTTTTTCCATAAATGCTGGTACTCTTTTGGGATTGGGAATAACTCCTTATATTACTGCTTCCATTGTAGTGCAATTTTTGCAAAAGCTCCTTCCTATTTGTCGTGAATGGAAAGACCAAGGACAAATGGGAAAACGCAAACTTAATCTTTTAACACGTAGTCTTGCCTTATTGTTTGCTTTTGGGCAATCTTTTGCTTTTTTGAACAGTTATTCAAAACTTTTAGTCACATCAATAAGTGCAATCCAACTGTTTTTGTTAGCTTTAATTGCTACTGCAGGAGTTGCTATTTTAATTTGGTTTGCTGACCTTATCAATTCCAAAGGTATTGGAAATGGGACTTCTATTTTAATTGTTGTTTCGATGAGCCACAGCCTAATTAATCTATTTGCAAATTTGAACAAATCATATTTATCTCAAAATAATTTTTTAACTTTAAAAACTTTTAATTTTGCATGTATTGTTCTTTTGCTTCTTTTATTTTTAATTTTTACTGTAGTTTTGCAAATAACATCTTTAAAAATACCTATCAATTATGCGCGCAATCAAGCGCAAGGCAAAAGCTACATTCCATTAAAAATTAATAGTGCAGGAGTTATGCCAGTTATTTTGGCATTTGCTTTATTGCAACCTTTCCAGATGTTAGCAGGAGTTATTGGGAATGACAAATTGACTATGTATGTAAATTTTTTTACCAACACCAATTCTTTTAATAACCAAATTAATTTTTTTGCTATAGGATTTTTAGTCTTGTTAGTGATTGTTCTTTCTTTCTTTTCTGCTTTTATGAATGTCAATCCTGAAGATATTTCAGAACATTTATCTAAACAAGATGCATATATTGCAGGTTTAAGACCAGGTGAACAAACTACTCGTTATTTAGCTAATACCTTATTTAAAATCACCGTTTTAGGAACTGTTTTTATTGTTGCTCTTGTTGTAACACCTATTCTTATGGAACATTTTTTAAGTTTGAAAGATATGAAATTAGGAGGAACCAGTTTGCTTATTATTGTTAGTGTAGCCCTTGAAACTATCCAACGCATCAAAGCTACTGCCAACAAAAAAGAATATCAAAAATTATTTTAATTAAGTAAACAAGACAACATGATACTAATAT	AAAAAATCCAAAAAAAATAAAATAATGGGAAGGAATAACTATGGAAACCAAAAACGCCAAAGCGATTGCTAGAAAAGTTTCAATCGCCCCTCGAAAAGCACGTTTAGTTGTTGATTTAATTCGAGGAAAAAATATTGCACAAGCTCAAGCAATTTTAACTTTTACCCCTAAAGTAGCTGCTCCCGTTATTTTAAAACTTTTAAACAGTGCTGTTTCCAATGCTGTTAATAATTTAAAATTAAACCGCGAACAACTTTATGTTAAAGAAGTTTTTGTTAACGAAGGTTTGCGTTTAAAACGTATGTTTCCAAGAGCTAAAGGTTCTGGTGATATGATTAAAAAAAGAACCAGCCACATTACTTTAGTAATAACTTCTAGCACAAACTTGCAAACATCAAAGGAGGAAGAACAAAGTGGGTCAAAAAACTAATCCTAACGGCTTAAGATTAGGCATTATTAGAACTTGGGAATCTCAATGGTTTGTTAATGATAAAGAAATTCCTAATTTAATTAAAGAAGATTTTTTAATTCGTAAACTAATTAATAATTTTGCTAAAAAAAGCGCTATCAGTCAAATTGACATCGAACGCCTAAAAGAAAAAAATAAAAACAGTATCACTATTTCTGTCCACACCGCTAAACCAGGCGTTATTATTGGAAAAGACGGCGATACACGCAACAAATTAGTTGCCAAAATAAAAGAGCTTACCCAAAAAGATGTTAATCTTAACGTTTTAGAAGTTAAAAACTCTGATAAAATCGCTTTATTAATTGCTCAAAATATGGCTGAACAACTAGAAAATCGTATGTTTTTCCGCCGTGTTCAAAAAATGGCAATCCAAAAAGCCCTAAAAGCAGGTGCTAAAGGAGTAAAAACTTTAATTTCTGGTCGTTTGGGTGGTGCTGAAATAGCTCGAAGCGAAGGACATGCTGAAGGCAGAGTTCCTCTACACACTCTAAGAGCAGACATCGATTACGCTGCTGTTGAAGCTCACACTAATTATGGAGTTTTAGGAATTAAAATATGGATTTTCCACGGTGAAGTTTTACCAGGACAAACCATTCTAGACACTAGAAAACCGTTTGCTTCCCAATCTTCTAACAATCCTAATATACGTT	ACTATGCTCACGTTGACTGTCCTGGACACGCTGATTATGTTAAAAACATGATTACTGGTGCTGCTCAAATGGATGCTGCTATTTTAGTTGTTTCTGGTGCAGATAGTGTTATGCCTCAAACTAGAGAACACATTTTATTAGCACGACAAGTTGGTGTTCCAAAAATTGTTGTTTTCTTAAATAAATGTGATCTTTCCCCAGACGAACAAATTTTAGAATTAGTAGAAATGGAAGTTCGTGAATTATTATCTCAATACGATTTCCCAGGAGATGAAATTCCTGTTATTAGAGGATCTGCATTAAAAGCTTTAGAAGGTGACGCTCATTACGTTGCTCAAGTAAATGAATTAATCGAAACTCTAGACACCTACATTGAAGACCCAATGCGTGAAGTTGATAAACCTTTCTTAATGCCTGTAGAAGATGTT
PPT3	AAAAGAGGGTTTCATAATTTTAATCAAGTTAAATATCATATAGTTAATTTGAAAGATTTAGAAATATTTGAAAATGGCACTTTAGTAACAAAAGAATTATTATTGAAAAAGAAAATAATTAAAAACGATAAATTACCTGTTAAGATTTTATCTAAAGGCGAATTTACTAAAAAAATAATTGTTCAAGATTGTAAAATGTCTCAGAAGGCAAAAGATATTATTGATAAAAATGATAATAGTAATACATAGAAATAATAAGGTAATTTTATGATGTTGAAATTGATTAGTTTTTTAGAAGATAGAAAAAAAATAATTAAAAAATGTTTTTTTACTTTGTTTCTTGTTTTTATTTATGTTATTGGTACTAAAATTTTTATCCCTTTATTAGGTGAAGATTTTTATTCGGTTTTATCATTAAAAAAAACATCTAGCGAAAATGATTTTTTGTTTTCAAATAATTCTTGTTCTCTTTTAGCATTAGGTGTTATCCCTTATGTTACTGCTTCTATAATAATTCAATTTGCTCAAAAAGTTTTTCCTTTCTTTCAAGAATGGCAAAAACAAGGAGATAAAGGTAAGTATAAAATTAATATAGTAACTAGAGTTTTAACAATTTTAATTGCTATAGCTCAAAGTTTTTTAATGTTACAACAAAATCCGATTTATTTGGCTAAATTATTAACGATAAAATACGAAGATTGGATTGTATTTCATTTTATTGTTATTTTCTTTTCAGTAGTTGGTGTTTTTATTTCTATTTGGTTAGCTGATTTAATAACTTCTAAAGGAGTAGGCAACGGGATTTCTCTTTTAATAGTTATAGGGATAATTGATAAATTATTTAATACTTTTAAGTATTTATTAGATTACAATAATCCGTTTTTGCATCAACGTTTGTTAGTATTATTTTCTTTTTTTATTTTGTTAATATTAACTATTTATTTATCTTTAGCTTATTTAAAACTTCCTATTAATTATGCTGTTGACCAACATAATGATAAAATAGATAATCATATTCCTTTTAAATTAAATACATCTGGTGTTTTACCGATTATTATTGCTGATACTTTTTTAAATATAACTAATTATATAAAAATTTTTTTTCCTAAATCCGTTAATTTTATTAATTTTTTTATTGACTCACGTTCTTATTTAGGAATATATTTTTTTGTTTATCTTTTATTAGTTATGTTGTTTTCTTTCTTTTCATCTTTTATGACTATTAATCCTACGGAAATTTCAGAACATTTATCGAAACAAAACGCTTATTTAAAAGATGTTCGACCAGGCTTACCTACTGTTAAAAAATTAACTTATGAAATATTTAAATTAACTTGTATTGGGAGTTTTTTTCTAACTTTGTTAGCTGCTGCTCCTGATGTAATTAGTTTTTGTTTTAATAATGAAATATCTCGGAATATTAAATTTGGCGGAACTAGTCTTTTAATTGTTGTTGGCGTTGCTATAGAAACTATACAAAATATTAAAAAAGAAAAGAATGTAAAAAATACTTATAGTAAAATTTTTTAAATTTACAAAAAGTGAATCATATATAAATTAGAGGTTATTTATTTTTTATGTTATCAATTAAATCCTCTCATGAAATTACTATAATGAGAAAAGCAGGTCAAATTTTATCCTGCATAAGAACAGAATTATTAAATTTTTTAAAAATAGGTATTTCTACTTTTGAATTAGATATGATCGCTTTCGAATTAATGAAAAAAAATGGAGT	ATGGTGGGTCATAAATTAGGAGAATTTTCGCCTACTCGTGTTTTTCGTGGTCATGCTAAAGGTGATAAGAAAAATCAAAAAAAATAATTTAAAGAAGGTATTTATATATGAAGGTTAAAGCGGTAGCAAGTCAAATACCTGTTACGCCTAGAAAAGTGTGTTTGGTTGTTGATTTAATTCGTGGTCAAAAAATTAAAGAAGCTGAAGCTATTTTAACTTTAAATTCAAAATCAGCTGCTCCTATTGTTTTGAAATTGTTAAAAAGTGTGGTAGCTAATGCTGTTCATAATTTTAATTTAAATAAAGATGATTTATATGTAGAAGAAATATTTGTTAATGAAAGCATTTCTTTACCTCGTTTATTTCCTAGAGCTAAAGGAAAGACAGATAAAAGAAAAAAGAGAACTAGTCATATCAAAATATTTGTTTCTAAATATAAAAAAAAAGAAACTCAGGAGATATAATATAAATGGGTCAAAAAAGCAATCCTAATGGTTTAAGATTAGGAATTATTCGTACTTGGGAATCTAAATGGTATGCTGAAGATAAACAAGTTCCTTCTTTGGTTTGCGAAGATTTTCAAATTAGAAATTTAATTAAAAATCATTATCCTAAAGCAACTATTGCTCAAATAGAAATAAAACGTTTAAAAAAAACGAATGATGAAGTTATTGAGATCGAACTATATACTTCTAAAATAGGTTTGATTCAAGGTCCAGATAATAAAAATAAAAATAGTTTAGTTAGTAAAATAGAAAATTTAATTAAAAAGAAAATTCAAATTAATATTTTTGAAGTAAAAGCTGTTAATAAAATTGCTGTTTTAGTAGCTCAAAATATAGCAATACAACTACAACAAAGAGCTTTTTATAAAGCTGTTCAAAAATCAGCTGTTCAAAAAGCTTTAAGAAGCGGTGTTAAAGGTATTAAAATTATTATTACAGGTCGTTTGGGCGGAGCTGAAAAAGCTAGACAAGAATCTATTTCTATGGGCGTCGTTCCTTTAAACACTTTGAGAGCTGATATTGATTATGCTTGCGAAGAAGCTCATACTACTTATGGCGTCTTAGGGGTTAAAGTTATTATTAATCATGGTGAAGTTTTGCCTAATAAGACTATTTCTGATACTAGACAAATATTTGCTTCTCAATATGATAATAAAAAACATTTT	TATGCTCATGTAGATTGTCCTGGACACGCAGATTATATTAAAAATATGATTACAGGAGCTGCTCAAATGGATGCAGGTATTTTAGTAGTTTCTGCTGTTGATGGTGTAATGCCTCAAACAAGAGAACATATTTTACTTGCTAAACAAGTAGGAGTTCCTAAGTTATTAGTTTTTTTAAATAAATGTGATATGATGGACGATGAAGATATTTTAGAATGTATAGAATTAGAAATTAGAGATGTTTTATCTTCTAATGGTTTCAATGATCCTAATATACCTATAATTAGAGGTTCTGCTTTGAAAGCTATAGAAGGCGATTCTAAATATGTTCAAAGTATACAAGATTTACTTGATGCTTTAGATACATATATTGAAGATCCAGTACGTGATTTAGATAAACCTTTTTTAATGCCTATAGAAGGTGTTAT
PPT4	AATTCCTAAAAGAGGATTTCATAACTTTTCACAAAAAAAATATGCTATTATTAACTTAAAAACTTTGCAAAATTTCTTAGAAGATAGTATTATTACTCCTCAGTTGTTATTAGAACAAAAAATTATTAAAAATAAATTAGCTGGTATTAAAGTGTTGGCTCAAGGGACTTTAACTAAAAGATTAGTCATCAAAGCTTCTAAATTTTCTCGTCAAGCGGAAGCTTGTATTTTAGCAGTTGGTGGAAAAATAGAGGTTATTTAAGATGAAAAGCAAAATAAAATTAATTTTAGGCAATCCAAAATTGATTTTTCAAATCTTATTTACTTTTTTTATTATTTTTCTTTTTAAGTTAGGTTCTGCTTTGCCTTTGCCTTTTATTCCTAAAGAATTTAAACTTTTAAAAATTTTAGGATTGATAGATGCTGCCCCATCAAAACTTTTTGGTTTAGGCATTTATCCTTATATTACAGCTTCTATTGTAGTTCAATTTTTACAAAAACTTTTACCTATTTGCCGTGAGTGGAAAGAACAAGGCCAAATCGGTAAACGTAAACTTAACCTTTTAACTAGGGCTTTAGCTTTATTATTTGTTTTTGGACAAACTTTTGGAATGATTCAAAAAACAAGTGATTCATTGGCAGTTTGCTTTTTGATCCCTTTAATTGCCGCTGCTGGATGTGCTATTTTAATTTGGTTTGCTGATTTAATCAACTCTCAAGGAATTGGTAATGGAACTTCTATTTTAATTATGGCCTCAATGAGTAATAATTTAATTGACTCTTTAAAAGAAATAAAACAAAATTATTACGACAATTTATTTACCAACAATTTTGACCCAAAATTGCTAACACAATTTATTTTAATTATCTTAGTATTGCTTTTATTTTTAATTGTAACTGTGATTGTCCAAATTACTTCTTTAAAAATACCAGTACAATACGCTCGCAATCAAAGTCCATCAAAAAGTAATAGTTATATCCCTTTTAAAATTAACACTGCTGGAGTAATGCCTGTAATTTTGGCAAATGCCTTGATGCAACCTTTCAAAATGTTAATCCCAATAATTAAAAATAATCAAGGTTTTGAGAATTTTGTCAATTATTTAACTAATATTGACATCGTTAATTTTGCTTTGAGCTTGCATATTTTATTAATTATTGTTTTTTCTTTTTTCTCTACTTTTATGAATGTTAATCCTGAAGATATTTCCGAGCATTTATCCAAACAAGATGCCTATATTGTTGGTTTTAGACCAGGAGAACAAACAACGAAATACTTATCTTCTCTTTTGTTCAAAATCACCGTTATAGGGGCTGTTTTTTTAGTTACACTCGTCACTATGCCTTACTTGATGAATAAAGTTTTTCATTTAAAACATATGAAATTAGGAGGAACTAGTTTACTAATTATCGTTAGTGTTGCAATTGAAACTATTCAACGAATTATGACTACCGCTAACCAAAAAGAATATGCAAAATTATTTTAATTCAGTAGACAAAAAAACTATGATATTAATATTATTAGGACCTCCAGGAATTGGTAAAGGCACTCAAGCGGCGTTTTTATCAGACCTTTTAAAAATGCATCATGTTGCAACAGGTGATATTTTTCGCAAAAATTTTAAAGAAAAAACCCAATTAGGTAAAGAAAGTAAAAAA	TTGTAGGTCACAAAATCGCTGTTTATAATGGAGCGAACATATTCCTGTTACATTACAGAAAACATGGTCGGTCATAAATTAGGAGAGTTTTCTCCTACCCGCACTTATCGTGGACATAACAAAAAAGACAAAAAAATTCAAAAAAAATAAAATAATGGGAAGGAATGACTATGGAAACTAAAAACGCTAAAGCGATTGTTCGTAAAGTTTCAATCGCCCCTCGAAAATCCCGTTTAGTGATTGACTTAATTAGAGGAAAAAATATCGATCAAGCTCAAGCCATTTTAACTTTTACTCCTAAAGTAGCTGCTCCCGTTATTTTAAAACTTTTAAATAGTGCTGTTGCAAATGCTTTAAATAATTTAAAATTACAACGTGAACAACTTTTTGTCAAAGAAGTTTTTGTCAACGAAGGCATACGTTTAAAGCGTATGTTTCCGAGAGCTAAAGGGTCTGGTGATATGATTAAAAAAAGAACTAGTCACATCACTTTAGTATGGCTGCTAAAAACTTGCAAACATCAAAGGAGGCGAACAGTGGGTCAAAAAACTAATCCTAATGGTTTAAGATTAGGTATTATTAGAACTTGGGATTCTCAATGGTGTGTCAATGACAAAGAAATACCAGCTTTAATTAAAGAAGAATTTTTAATTCGCAAAATAATTAATCAATTTGGTAAAAAAAGCGCTATTAGTCAAATTGAAATACAGCGTTTAAAAGAAAAAACCAAAAATCGGATCACAATTTCAATTCATACCGCCAAACCAGGAATGATTATCGGTAAAGAGGGTGAAACTCGCAATAAAATAGTAGCGCAATTAAAAACATTAACTGGCAAAGACATTAACTTAAATATTATAGAAGTTAAAATCCCAGATAAAATAGCTTTATTAGTAGCACAAAATATTGCTGAACAATTAGAAAACCGAATGAAATTTCGTCGTGTTCAAAAAATGGCGATCCAAAGAGCCCTTAAATCTGGCGCTAAAGGGATTAAAATTTTAATTTCTGGTCGTCTTAATGGCAATGAAATAGCGCGTAGCGAAGGCAATGCTGAAGGTCGTGTTCCTTTGCACACTTTAAGAGCAGATATTGATTATGCTGCCTAGACACACACATTATGGTGTTTTAGGGATCAAAGTCTGGATTTTTCACGGTGAAGTTTTATCCGGTCAAACCAT	TATGCTCACGTTGATTGTCCAGGACACGCCGATTATATTAAAAACATGATTACTGGTGCTGCTCAAATGGATGCCGCTATTTTAGTAGTTTCTGGCGCAGATAGTGTTATGCCTCAAACTAGAGAACATATTTTGTTAGCTCGCCAAGTAGGGGTTCCTAAAATTGTTGTTTTCTTAAACAAATGTGACCTTTGTCCGGATGAAGAAATTTTAGAATTAGTTGAAATGGAAGTCCGTGAATTATTATCTAAATATGATTTTCCGGGCGATGATACCCCTATTATTAGAGGTTCTGCGTTAAAAGCTTTAGAAGGTGACAAACATTACATTGCACAAGTTAACGAATTAATCAACGCTTTAGATTCTTACATTGAAGATCCAGTGCGTGAAGTTGATAAACCTTTCTTAATGCC
